# The impact of internet use on physical activity among Chinese older women: the mediating role of social support and intrinsic motivation

**DOI:** 10.3389/fpubh.2026.1821869

**Published:** 2026-06-12

**Authors:** Jinyu Lin, Ping Wang, Juhua Wu, Wei Liu, Guan Yang

**Affiliations:** 1 School of Physical Education, Guangxi University of Science and Technology, Liuzhou, China; 2 School of Physical Education, South China University of Technology, Guangzhou, China

**Keywords:** internet use, intrinsic motivation, older women, physical activity, social support

## Abstract

**Background:**

Internet use has brought tremendous convenience to the lives of older women. Although previous studies have examined the association between internet use and physical activity among older adults, empirical research on the psychosocial pathways linking internet use to physical activity among older women remains limited. To bridge this gap, this study examined the mediating roles of social support and intrinsic motivation in the association between internet use and physical activity among older women.

**Methods:**

A total of 976 older women from 10 provinces in China completed a structured questionnaire. This study used correlation analysis and multiple linear regression to examine the association between internet use and physical activity. The SPSS PROCESS macro was employed to test the mediating roles of social support and intrinsic motivation in this association.

**Results:**

The results indicated that social support and intrinsic motivation mediate the association between internet use and physical activity among older women. Moreover, social support and intrinsic motivation sequentially mediate this association.

**Conclusion:**

The study concludes that internet use was associated with physical activity through social support and intrinsic motivation. These findings suggest that social support and intrinsic motivation may be important psychosocial pathways linking internet use to physical activity. Supporting appropriate internet use and digital engagement among older women may help promote physical activity and support healthy aging. Digital or remote physical activity programs for older women should incorporate social support and motivational elements. Such strategies may have indirect implications for musculoskeletal health promotion in later life.

## Introduction

Population aging has emerged as a pressing social challenge in most countries worldwide, with China confronting a particularly profound demographic shift. According to the National Bureau of Statistics of China, by 2025, China’s population aged 60 years and older will reach 323 million, accounting for 23% of the total population (1). Among this demographic, older women constitute 52.2% (2), reflecting a higher proportion of women than men in later life. As population aging deepens, the feminization of the older population is projected to intensify, positioning older women as a socially consequential group (3). Due to longer life expectancy, older women face numerous challenges associated with population aging. In the context of aging, the most prominent problem is the health disparities caused by gender differences. Across all societies, older women are more vulnerable to discrimination and social neglect (4). Throughout the life course, they face societal inequities in education, employment, income, status, and access to other resources. After entering old age, the intertwining of gender discrimination and ageism traps them deeper in situations of loneliness, dependency, poverty, illness, marginalization, and social exclusion (5). Under the combined influence of various adverse factors, older women’s health problems have become particularly serious. The World Health Organization’s World Report on Aging and Health indicates that older women face higher disease prevalence and poorer health outcomes than men (6). Similarly, data from the 2023 China Longitudinal Aging Social Survey (CLASS) indicate that older women’s physical health is worrisome and warrants greater attention (7). Consequently, improving the health of older women has become an unavoidable priority in China’s national strategy to address population aging. More importantly, for older women, regular physical activity also helps maintain musculoskeletal health, including bone health, muscle strength, mobility, balance, and functional independence in later life (8, 9). Therefore, increasing physical activity levels among older women is not only important for healthy aging in general but also crucial for promoting musculoskeletal health.

In order to address the health challenges of aging, in 2021, the Central Committee of the Communist Party of China and the State Council issued the “Opinions on Strengthening Aging Work in the New Era”, which identifies physical exercise as one of the key measures to counter population aging and encourages older adults to actively engage in physical activity to improve their health. Numerous previous studies have demonstrated that physical exercise can effectively improve physical function (10), delay cognitive decline (11), and reduce disease risk (12, 13) among older women. Therefore, increasing older women’s participation in physical activity to support the construction of Healthy China has become an important issue for scholars. In addition to daily communication and leisure activities, Internet use may provide older adults with digitally supported or remotely delivered exercise guidance, home-based physical activity support, and online opportunities for physical activity promotion. Recent evidence indicates that home-based exercise interventions delivered via digital health platforms and telehealth-based exercise interventions can improve physical function in older adults, and digital physical activity interventions may also increase physical activity participation among this population (14–16).

With the rapid development and popularization of the internet, the proportion of older adults who surf the internet continues to rise, and the internet (including mobile Internet) has gradually become an important tool for obtaining information, news, entertainment, and leisure in their daily lives. According to the 57th Statistical Report on the Development of the Internet in China released by the China Internet Network Information Center (CNNIC), as of December 2025, China had 1.125 billion internet users; when divided by age group, the internet penetration rate among people aged 60 and older reached 53.7% (17). Previous research has demonstrated that internet use can alleviate depression and loneliness (18), enhance physical and mental health (19), foster social participation and improve quality of life (20), and also facilitate access to information related to physical activity (21) and health among older adults. The information that older adults obtain online can equip them with the knowledge and skills needed to engage in physical activity, boost their motivation, and improve their physical and mental well-being. Furthermore, although physical activity is crucial for musculoskeletal health in later life, little research has explored how digital engagement may support this behavior through relevant mechanisms; these findings that could inform the development of future digitally supported or remotely delivered interventions for older women. Against the backdrop of an aging population, the internet has become a significant factor influencing older adults’ engagement in physical activity.

Prior research has focused on the relationship between internet use and physical activity among older adults. Cui et al. (22) found that the frequency of digital use was significantly associated with several healthy lifestyle behaviors among Chinese older adults, including exercise. As a key external resource, social support can enhance older adults’ motivation to engage in physical activity when using the internet by providing material, emotional, and informational assistance (23, 24). Meanwhile, as a core internal factor driving individual health behaviors, intrinsic motivation is a key psychological determinant of sustained physical activity among older adults (25). Although the overall association between internet use and older adults’ physical activity has been preliminarily verified, empirical evidence focusing specifically on older women remains insufficient. In particular, the pathways and mechanisms through which internet use influences physical activity among older women have not been systematically examined.

On this basis, this study focuses on the impact of internet use on physical activity among older women in China, with particular emphasis on testing the mediating effects of social support and intrinsic motivation. In doing so, the findings provide theoretical and practical insights to promote participation in physical activity among older women and advance healthy aging. They also offer valuable guidance for digitally supported or remotely delivered interventions targeting musculoskeletal health in later life.

## Literature review and research hypotheses

### Internet use and physical activity

Physical inactivity among older adults is a major challenge to healthy aging (26). Internet use may be positively associated with physical activity by providing exercise-related information, health knowledge, online fitness videos, activity reminders, and self-monitoring resources. First, as a platform characterized by convenience, wide accessibility, and diverse content, the Internet offers considerable potential to support the health and well-being of older adults (27). Increasingly, older adults are engaging with internet platforms not only for real-time monitoring and personalized feedback on physical activity, but also to access evidence-based behavioral interventions designed to support sustained health behavior change. Empirical studies have demonstrated that internet use is associated with more sustained engagement in physical activity among older adults (28). Internet use offers several key advantages, including improved accessibility, reduced burden on healthcare providers, and high scalability for large-scale implementation. Moreover, these platforms can assist older adults in setting goals, developing action plans, monitoring their behavior, and receiving feedback. These functions have been associated with increased physical activity levels in the short term (29). Recent research indicates that web- and mobile-based interventions can encourage older adults to engage in physical activity, but this requires designing interfaces and content suitable for older adults, employing tailored behavior change techniques, and promoting family involvement (30). Given the close connection between sustained physical activity and musculoskeletal health in old age, understanding the association between internet use and physical activity among older women may help inform the development of digital or remote strategies to promote musculoskeletal health. Based on these potential benefits, we hypothesize that the direct association between internet use and physical activity would be positive. Specifically, internet use may reduce barriers to accessing information and provide flexible resources for home-based exercise, community activities, and self-monitoring. However, this association may also be complex, as excessive screen time may displace physical activity and contribute to sedentary behavior.

Second, internet use may also be indirectly associated with physical activity through psychosocial pathways. Previous studies have shown that internet use is associated with reduced depressive symptoms, improved physical and mental health, and increased overall life satisfaction (31). For example, Heo et al. (32) suggest that older adults may use online platforms to access health-related information, expand their social networks, improve their interpersonal communication skills, and increase their opportunities for social participation. Research also demonstrates that more frequent internet use is associated with more frequent daily physical activity and more weekly sessions of moderate-to-vigorous physical activity (33). However, this relationship may vary depending on differences in digital access, digital skills, and usage patterns. The digital divide may limit older adults’ ability to benefit from digital health resources (34), while excessive or passive screen time may displace physical activity and increase sedentary behavior (35). In summary, previous studies have shown a positive correlation between internet use and physical activity among older adults. However, existing research has tended to focus on behavioral outcomes or intervention effectiveness, with insufficient exploration of the psychosocial pathways linking internet use to physical activity among older adults. Furthermore, such research typically treats older adults as a homogeneous group, whereas relatively little attention has been paid to older women. Less is known about how these mechanisms can inform digital or remote physical activity strategies for older women. Therefore, this study focuses on older women and examines the psychosocial pathways through which internet use is associated with physical activity, addressing gaps in the understanding of psychosocial pathways and insufficient attention to specific populations in existing research.

### The mediating role of social support

Social support is widely recognized as an important psychosocial factor associated with physical activity among older adults. Current research indicates that different forms and sources of social support have varying effects on physical activity. Among older adults, social support from significant others (e.g., family, friends) has been consistently associated with higher levels of physical activity participation and sustained adherence (36). Older adults who receive emotional, appraisal, and companionship support from family and friends are more likely to be physically active. For example, Ranby and Aiken indicated that among married older women, higher weekly physical activity levels are associated with husbands’ involvement in their activity goals and providing positive encouragement for their progress (37). Moreover, older women who receive encouragement and accompaniment from family and friends during physical activity are more likely to engage in higher-intensity moderate-to-vigorous physical activity (21, 38). Beyond the support of significant others, friends’ support is particularly crucial, as most physical activities take place within peer social environments (39). For instance, O’Brien reported that among women aged 70 years and older, support from friends was the strongest correlate of physical activity participation (40). Nevertheless, there is still no consensus on the relative importance of support from family and friends. This inconsistency may reflect not only differences in the sources of support but also in the specific forms of support examined, such as emotional support, companionship, and practical assistance. In addition, factors such as gender, marital status, and cultural background may influence how older adults access, perceive, and respond to physical activity-related support. These findings suggest that social support should not be treated as a uniform construct with a single pathway to physical activity, as its effects may vary according to the source and type of support, as well as gendered social roles.

Existing research has documented a positive association between internet use and perceived social support, suggesting that digital engagement may serve as an important resource for maintaining and expanding social connections in later life. First, internet use may create a novel space for social interaction, enabling individuals to maintain existing relationships and establish new social connections, thereby enhancing perceived social support, social identity, and a sense of belonging (41). In addition, empirical research on older adults suggests that internet use has been associated with lower social support deficits (42), and online engagement is associated with higher perceived social support (43). For instance, Shen et al. (44) found that older adults’ internet use may increase their interactions with exercise partners, thereby strengthening social support and encouraging more active participation in physical activity. Overall, existing research has examined the relationships among internet use, social support, and physical activity. However, social support is often viewed as either a direct determinant or a contextual factor of physical activity, rather than a mediating mechanism through which digital engagement influences behavior. Therefore, it remains unclear whether social support serves as a psychosocial pathway linking older women’s internet use to physical activity. Clarifying this pathway may help inform digital physical activity interventions that integrate social connection with interpersonal support.

### The mediating role of intrinsic motivation

Self-Determination Theory (SDT) has been widely applied across fields such as education, work, and exercise research to examine the relationship between motivation and specific behaviors or activities. SDT views motivation as a continuum ranging from amotivation and controlled regulation to more autonomous forms of motivation, including identified regulation, integrated regulation, and intrinsic motivation. Research based on this framework indicates that individuals may exhibit different forms of motivation and varying degrees of autonomy in specific contexts and activities (45). According to Deci and Ryan, intrinsic motivation is the most autonomous form of motivation. It reflects engagement in an activity for its inherent satisfaction, such as interest, enjoyment, or a sense of competence, rather than for instrumental reasons (46). Although SDT encompasses various forms of autonomous motivation, this study focuses on intrinsic motivation because, for older women, enjoyment, interest, and personal satisfaction may be particularly important in sustaining daily physical activity. In later life, older women’s participation in physical activities is often no longer primarily driven by formal performance goals but rather depends more on whether the activity is experienced as meaningful, enjoyable, and personally rewarding. From this perspective, intrinsic motivation is a theoretically appropriate and conceptually focused mechanism that links internet use, social support, and physical activity.

The SDT further posits that when the three basic psychological needs of autonomy, competence, and relatedness are satisfied, more self-determined forms of motivation and health-promoting behaviors are likely to emerge (47). Recent research has further clarified that a supportive environment can be understood as specific behaviors aligned with these three needs, thereby providing a more precise basis for explaining how the external environment fosters intrinsic motivation (48). Conversely, when these needs are frustrated, individuals may develop controlled motivation, which can undermine sustained engagement and psychological well-being (49). Researchers indicate that among adults, self-determined motivation, specifically motivation linked to enjoyment, competence, and social relationships, is positively associated with physical activity participation (50). Similarly, other researchers have found that among older adults, stronger intrinsic motivation is associated with higher level of physical activity (51). In particular, when older adults experience enjoyment, a sense of accomplishment, and social connections from physical activities, they are more likely to develop intrinsic motivation for physical activity.

In this context, internet use may serve as a contextual resource for fostering intrinsic motivation. By expanding access to health information and providing tools for self-monitoring, feedback, and guidance, internet use may strengthen individuals’ sense of autonomy and competence regarding physical activity (52). At the same time, online media may also shape individuals’ awareness, attitudes, and motivations toward physical activity participation. When older women access online fitness information, it may enhance their perceptions of capability and understanding of the benefits of physical activity, thereby supporting intrinsic motivation, which may be associated with more frequent participation in physical activity. In summary, although existing research has widely applied self-determination theory (SDT) to the field of physical activity, little is currently known about whether intrinsic motivation constitutes a specific psychological pathway linking internet use to physical activity among older women. Clarifying this pathway may also help inform the development of digital physical activity interventions that incorporate personalized feedback, autonomy-supportive guidance, and engaging activity content to enhance older women’s intrinsic motivation. Therefore, this study aims to examine whether intrinsic motivation mediates the relationship between internet use and physical activity among older women.

### The chain mediating role of social support and intrinsic motivation

Social support and intrinsic motivation are two key psychosocial factors associated with older women’s participation in physical activity and may be important for both the initiation and maintenance of this behavior. Empirical studies consistently demonstrate that older adults’ willingness to engage in physical activity is often shaped by the combined role of external resources and internal motivational processes (24). However, relatively little attention has been paid to how these external and internal factors operate sequentially, particularly in the context of internet use.

Social support can be viewed not only as an external facilitating factor but also as a vital interpersonal resource that primarily fulfills the need for relatedness and may also support competence and autonomy (52). Specifically, support from family members, friends, and community members, such as verbal encouragement, positive feedback, emotional support, and practical assistance, may strengthen an individual’s sense of social connectedness, enhance confidence in their physical abilities, and foster a greater sense of autonomy in exercise participation, thereby supporting intrinsic motivation for physical activity (53). Previous research has shown that supportive exercise environments, especially those that foster a sense of belonging, competence, and autonomy, may be associated with stronger intrinsic motivation for physical activity (54). In addition, social support may initially provide encouragement, companionship, feedback, and practical assistance, thereby enhancing intrinsic motivation and supporting continued participation in physical activity. Intrinsic motivation can be viewed as a key psychological pathway through which a supportive social environment is associated with sustained physical activity (55). Overall, these studies suggest a potential psychosocial pathway linking internet use and physical activity among older women: internet use may be associated with social support, which in turn may be associated with stronger intrinsic motivation and higher levels of physical activity participation. Although previous studies have examined internet use, social support, motivation, and physical activity separately, research integrating social support and intrinsic motivation into a chain mediation model remains relatively scarce, especially among older women in digital contexts. This sequential pathway may provide a psychosocial basis for designing digital or remote physical activity interventions that integrate social support with motivational enhancement. Therefore, this study aims to examine whether internet use is associated with physical activity among older women through a chain mediation pathway involving social support and intrinsic motivation.

**Figure 1:**
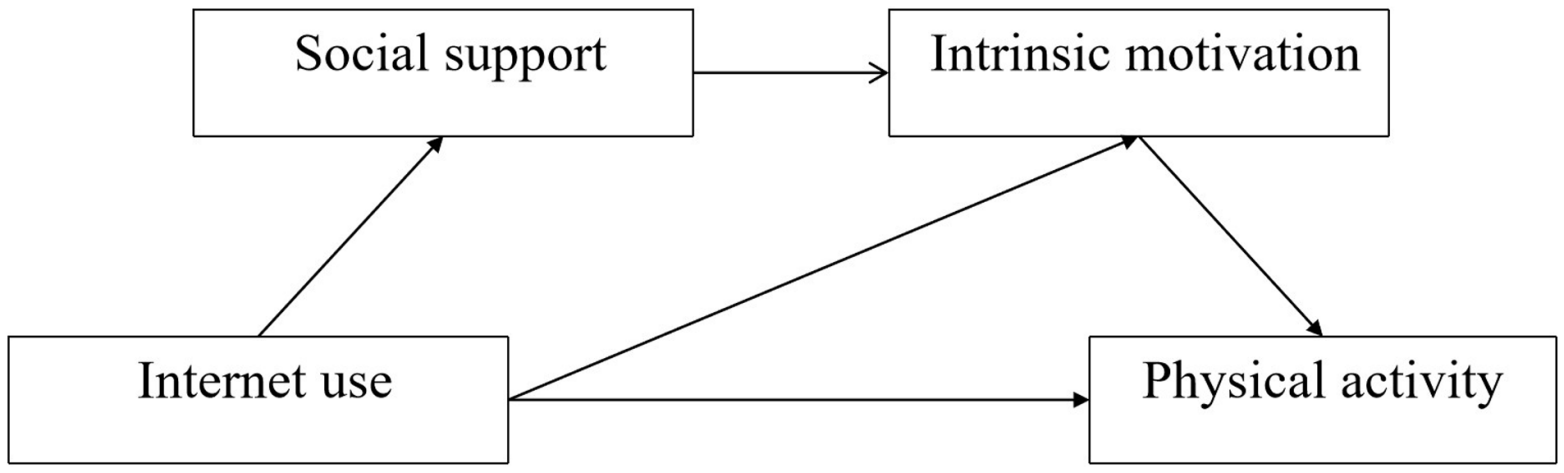
The hypothesized model.

Based on the aforementioned theoretical foundation and prior research findings, this study proposes the following research hypotheses (Figure 1):

*Hypothesis 1*: Internet use is positively associated with older women's physical activity participation.

*Hypothesis 2*: Social support mediates the association between internet use and older women's physical activity participation.

*Hypothesis 3*: Intrinsic motivation mediates the association between internet use and older women’s physical activity participation.

*Hypothesis 4*: Social support and intrinsic motivation sequentially mediate the association between internet use and older women's physical activity participation.

## Methods

### Sample and participants

This study employed multistage sampling to enhance sample representativeness. China’s eight major regions—namely, municipalities directly under the Central Government, the Northeast, the Northwest, the Central, the East, the South, the Southwest, and the Southeast were selected as the sampling frames and sampled in the order from macro regions to provinces to cities to districts. Additionally, to balance the proportions of urban and rural samples, an equal ratio was maintained within each zone during sampling. From November 25 to 30, 2019, administrators received training on the fundamentals of survey administration. The formal survey was carried out from December 1 to 31, 2019. Before administering the questionnaire, the investigators explained the purpose of the survey to the older women, assured them of strict data confidentiality, and emphasized that all data would be used for research purposes. As most rural older women have low educational attainment and have difficulty understanding the questionnaire content, the investigator read each question to the respondent one by one, allowing them to make their own subjective judgments and selections. Among the 1,008 older women invited to participate, all completed the questionnaire. After excluding invalid responses with similar or incorrect answers, a total of 976 valid samples were obtained.

### Measures

#### Dependent variable

The dependent variable in this study is older women’s participation in physical activity. According to the American scholar Kenyon’s definition of sports participation, it encompasses four levels: “cognition,” “emotional orientation,” “direct participation,” and “indirect participation” (55). In this study, sports participation is limited to the level of “direct participation,” that is, direct involvement in sports activities. In the questionnaire, respondents were asked, “Did you participate in sports activities per week?” The options were, in order: (1) “never,” (2) “1–2 times,” (3) “3–4 times,” (4) “5–6 times,” (5) “more than six.” For the purposes of this analysis, we combined and coded the responses as follows: “never” (=1), “1–2 times” (=2), “3–4 times” (=3), “5–6 times” (=4), “more than six” (=5).

#### Independent variable

The independent variable is internet use. According to a 2025 study by Shen et al. (44), we chose the question “Did you usually access the internet via mobile phone or computer?” to measure internet use in the questionnaire. Based on the respondents’ answers, a 5-point frequency scale was used for scoring, ranging from “never” (=1), “rarely” (=2), “sometimes” (=3), “often” (=4), “very frequently” (=5). A higher score indicates a higher frequency of internet use among older women.

#### Mediating variables

Social support was assessed using a scale developed by Xu (56). This scale consists of two dimensions: formal support (including government, social organizations, and communities) and informal support (including family, friends, and neighbors). A 5-point Likert scale was used, ranging from “Strongly Disagree” (=1), “Disagree” (=2), “Neutral” (=3), “Agree” (=4), “Strongly Agree” (=5). To enhance the scale’s applicability to older women in China, the researchers made targeted revisions prior to formal administration. Specifically, the wording of some items was adjusted to improve clarity and age appropriateness, and several new items were added to more accurately reflect older women’s social support experiences in daily life. The revised items were reviewed by experts in sports sociology, gerontology, and survey methodology to ensure content relevance and comprehensibility. Additionally, a pilot study was conducted with a sample of 300 older women to further refine the wording of the items. Based on these efforts, this study re-examined the reliability and validity of the adapted scale. The Cronbach’s *α* for this scale was found to be 0.971, and the Cronbach’s α for each dimension was found to be 0.968 and 0.963. The results of the confirmatory factor analysis were *χ*^2^/df = 3.329, TLI = 0.984, CFI = 0.994, RMSEA = 0.079, and SRMR = 0.015, indicating good reliability and validity.

Intrinsic motivation was measured using five items from the intrinsic motivation subscale of the Behavioural Regulation in Exercise Questionnaire (BREQ) by Mullan et al. (57). To enhance the applicability of the intrinsic motivation scale in the context of Chinese culture among older women, this study made appropriate revisions to the relevant items to better align with their language habits and exercise experiences. Firstly, the original English items were translated and back-translated by bilingual researchers, and discrepancies between the translations were resolved through repeated discussions to ensure semantic equivalence. Secondly, the adapted items were evaluated for content validity by experts in the relevant field, and a pre-survey was conducted with 300 older women to test the items’ comprehensibility and appropriateness, after which minor wording revisions were made. This study re-evaluated the revised version of the scale. The Cronbach’s *α* for this scale was found to be 0.826; the results of the confirmatory factor analysis were *χ*^2^/df = 5.469, TLI = 0.989, CFI = 0.998, RMSEA = 0.042, and SRMR = 0.010. Although the *χ*^2^/df value is relatively high, the remaining fit indices are all within the recommended thresholds. Given that the chi-square statistic is sensitive to sample size, this study considers the model’s overall fit acceptable.

#### Control variables

We included the following control variables: age, education level, monthly income, place of residence, and marital status. Of these, age (50–92 years). Educational attainment was categorized as follows: primary education or below (=1), junior high school (=2), senior high school (=3), university education and above (=4). Place of residence (1 = urban, 0 = rural). Marital status (0 = without a spouse, 1 = married with spouse).

### Statistical analysis

To examine the association between internet use and physical activity among older women and to assess potential psychosocial pathways, we conducted statistical analyses using SPSS version 27. First, descriptive statistical analyses were conducted on key variables, including physical activity, internet use, intrinsic motivation, and social support. The means and standard deviations were calculated to describe the distribution of the main study variables. Second, Pearson correlation analyses were conducted to examine the relationships among internet use, social support, intrinsic motivation, and physical activity. Pearson correlation analysis was used to assess the linear bivariate associations among the main continuous variables. All tests were two-tailed, and *p* < 0.05 was considered statistically significant. Third, before conducting regression analysis, the assumptions of linearity, normality of residuals, homoscedasticity, and multicollinearity were examined and found to be acceptable. Then, we used ordinary least squares (OLS) regression to estimate the associations among internet use, social support, intrinsic motivation, and physical activity, while controlling for age, educational attainment, monthly income, place of residence, and marital status. Since the dependent variable in each regression equation was treated as a continuous variable and the analysis aimed to examine the relationships among the study variables after adjusting for relevant covariates, OLS regression was considered appropriate. Finally, serial multiple mediation was examined using Model 6 of the PROCESS macro. Model 6 was chosen because the proposed theoretical framework assumed a sequential mediation pathway: Internet use is associated with social support, social support is subsequently associated with intrinsic motivation, and intrinsic motivation is associated with physical activity. The direct and indirect effects were estimated using 5,000 bootstrap samples with 95% confidence intervals. When the 95% bootstrap confidence interval does not include zero, the indirect effect is considered statistically significant.

## Results

### Common method bias

To assess potential interference from common method bias, this study employed Harman’s single-factor test to examine common variance in the obtained data. The results showed that the first factor from the unrotated exploratory factor analysis accounted for less than 40% of the total variance, indicating that common method bias was not serious in this study.

### Assumption checks for parametric analyses

The diagnostic tests indicate that the assumptions underlying Pearson correlation and ordinary least squares (OLS) regression were generally acceptable. Neither the scatter plot nor the residual plot indicates a serious violation of linearity or homoscedasticity. The normal P–P plot shows that the standardized residuals generally lie along the diagonal. Furthermore, the standardized residuals range from −2.158 to 2.914, indicating no extreme residual values. Multicollinearity was not a serious issue, as all VIF values were below 3. The Durbin-Watson statistic was 1.735, indicating no serious issues with the independence of residuals. Overall, these diagnostic results indicate that the data are suitable for ordinary least squares (OLS) regression analysis.

### Descriptive statistics and correlations

Table 1 presents descriptive statistics for the key variables. The mean age of the respondents was 60.53 years. Among older women, 63.7% had completed secondary education; 67.4% resided in urban areas, and 32.6% in rural areas; and 84.5% were married (with spouse). The average physical activity score was 2.70 (SD = 1.188). The mean social support score was 163.75 (SD = 38.53), internet use averaged 3.05 (SD = 1.275), and the mean intrinsic motivation score was 17.967 (SD = 4.147).

**Table 1:** Basic variable description statistics table.

Variable	Obs	Mean	SD	Min	Max
Internet use	976	3.05	1.275	1	5
Social support	976	163.750	38.525	51.00	255.00
Intrinsic motivation	976	17.967	4.147	5.00	25.00
Physical activity	976	2.70	1.188	1	5
Age	976	60.53	7.392	50	92
Education	976	2.405	0.984	1	4
Monthly incomes	976	2.94	1.528	1	5
Residence	976	0.674	0.469	0	1
Marital status	976	0.845	0.362	0	1

Table 2 illustrates the correlation matrix for the core variables in this study. Physical activity was significantly correlated with all variables, including internet use (*r* = 0.169, *p* < 0.01), social support (*r* = 0.267, *p* < 0.01), and intrinsic motivation (*r* = 0.321, *p* < 0.01); internet use was positively related to social support (*r* = 0.556, *p* < 0.01) and intrinsic motivation (*r* = 0.418, *p* < 0.01); social support was found to be strongly positively correlated with intrinsic motivation (*r* = 0.661, *p* < 0.01).

**Table 2:** Pearson’s correlations among variables.

Variables	1	2	3	4
1. Physical activity	1			
2. Internet use	0.169^**^	1		
3. Social support	0.267^**^	0.556^**^	1	
4. Intrinsic motivation	0.321^**^	0.418^**^	0.661^**^	1

*N* = 976. *p < 0.1, **p < 0.05, ***p < 0.001.

### Regression model testing

As presented in Table 3, all nested regression models show statistically significant improvements in model fit. Model 1, serving as the baseline specification, included only control variables to estimate their association with physical activity. Notably, education (*β* = 0.231, *p* < 0.001) is positively correlated with older women’s participation in physical activity. Model 2 examined the association between internet use and physical activity. The results showed that the regression coefficient for internet use was 0.128 and was significant at the 0.001 level. This finding indicates that internet use was positively associated with physical activity: older women who use the Internet more frequently exhibit higher levels of physical activity. Models 3 and 4 examined the roles of social support and intrinsic motivation in physical activity. In both models, internet use was not associated with physical activity (*p* > 0.05), indicating that social support (*β* = 0.008, *p* < 0.001) and intrinsic motivation (*β* = 0.067, *p* < 0.001) may mediate the relationship between internet use and physical activity among older women, providing critical support for subsequent tests of the mediating effect.

**Table 3:** Impact of internet use on physical activity among Chinese older women: based on OLS model.

Variable	Model 1	Model 2	Model 3	Model 4
Constant	2.308^***^ (0.382)	1.469^***^(0.433)	0.334(0.452)	−0.151(0.453)
Age	−0.002(0.005)	0.005(0.006)	0.009(0.006)	0.010(0.006)
Education	0.231^***^(0.047)	0.210^***^(0.047)	0.213^***^(0.046)	0.201^***^(0.045)
Monthly incomes	−0.001(0.031)	−0.005(0.031)	−0.003(0.030)	−0.007(0.030)
Residence	−0.076(0.057)	−0.051(0.057)	−0.045(0.055)	−0.027(0.055)
Marital status	0.041(0.061)	0.052(0.061)	0.050(0.060)	0.031(0.059)
Internet use		0.128^***^(0.032)	0.001(0.036)	−0.009(0.036)
Social support			0.008^***^(0.001)	0.003^**^(0.001)
Intrinsic motivation				0.067^***^(0.012)
*N*	976	976	976	976
*R^2^*	0.050	0.065	0.111	0.140
Δ*R^2^*	0.045	0.059	0.104	0.133
*F*	10.104^***^	11.224^***^	17.183^***^	19.752^***^

*p < 0.1, **p < 0.05, ***p < 0.001.

### Mediation effect analysis

The results in Table 4 indicate that the coefficient for internet use on social support was 0.556, with 95% confidence intervals (CI) that do not include 0. The coefficients for internet use and social support on intrinsic motivation were 0.621 (95% CI [0.564, 0.677]) and 0.073 (95% CI [0.016, 0.129]), and the 95% confidence intervals (CI) also exclude 0. Similarly, the coefficients for internet use, social support, and intrinsic motivation on physical activity were 0.011, 0.091, and 0.256, and the 95% confidence interval (CI) for the association between internet use and physical activity includes zero. In contrast, the 95% confidence intervals for the associations of social support and intrinsic motivation with physical activity exclude zero. These findings suggest that the association between internet use and physical activity among older women may be mediated by social support and intrinsic motivation.

**Table 4:** Path-coefficients of the mediating models.

Variable	*β*	BC95%LL	BC95%UL	*R* ^2^	*F*
Internet use → Social support	0.556	0.504	0.608	0.309	435.880^***^
Internet use, Social support vs. intrinsic motivation	0.073	0.016	0.129	0.4412	384.102^***^
Social support → intrinsic motivation	0.621	0.564	0.677		
Internet use, Social support, intrinsic motivation vs. physical activity	0.011	−0.061	0.083	0.108	39.3322^***^
Social support → physical activity	0.0914	0.005	0.178		
Intrinsic motivation → physical activity	0.2557	0.176	0.335		

Model 6 of PROCESS was employed to test the multiple mediating effects of social support and intrinsic motivation on the association between internet use and physical activity. From the total effects in Table 5, internet use was positively associated with physical activity (*β* = 0.169, 95% Bootstrap CI: 0.107, 0.231). However, the direct effect analysis showed that internet use was not significantly associated with physical activity (*β* = 0.011, 95% Bootstrap CI: −0.061, 0.083). Most importantly, social support significantly mediated the association between internet use and physical activity (indirect effect: *β* = 0.051; 95% bootstrap CI [0.0002, 0.101]). The indirect effect via intrinsic motivation was 0.019 (95% bootstrap CI [0.000, 0.039]), and the serial indirect effect via social support and intrinsic motivation was 0.088 (95% bootstrap CI [0.059, 0.121]). The total indirect effect was 0.158, accounting for approximately 93.5% of the total association. Among these three specific indirect effects, the serial mediation pathway via social support and intrinsic motivation showed the largest effect size, accounting for approximately 52.7% of the total effect. These findings suggest that the association between Internet use and physical activity among older women is primarily explained by psychosocial pathways rather than by direct mechanisms. Overall, the mediating effect consists of three paths: (1) internet use → social support → physical activity; (2) internet use → intrinsic motivation → physical activity; (3) internet use → social support → intrinsic motivation → physical activity. Collectively, these findings provide empirical support for Hypotheses 2 through 4.

**Table 5:** Mediating effects of social support.

Paths	Standardized coef.	Bootstrap 95%CI	
		Lower	Upper
Total effect			
Internet use → physical activity	0.169	0.1068	0.2307
Direct effects			
Internet use → physical activity	0.011	−0.0607	0.0828
Indirect effects			
Internet use → social support → physical activity	0.051	0.0002	0.1014
Internet use → intrinsic motivation → physical activity	0.019	0.0009	0.0391
Internet use → social support → intrinsic motivation → physical activity	0.088	0.0580	0.1213

## Discussion

Regular physical activity is closely related to mobility, balance, functional independence, and musculoskeletal health in older women. Therefore, identifying the psychosocial pathways related to physical activity may provide useful evidence for developing digital strategies to support physical activity participation and promote musculoskeletal health in this population. This study systematically examined the pathways through which internet use is associated with physical activity among older women in China, with a particular focus on the mediating roles of social support and intrinsic motivation. The results of this study show that internet use is indirectly associated with physical activity, primarily through a chain of mediating pathways involving social support and intrinsic motivation. Several important conclusions can be drawn from this research.

The first finding was that internet use was not directly associated with physical activity among Chinese older women, consistent with most previous studies. Unlike previous studies that have primarily focused on the direct link between Internet use and physical activity, this study further suggests that a deeper understanding of their relationship may be gained by examining the mediating roles of social support and intrinsic motivation. For instance, He indicates that there is no significant correlation between internet use and older adults physical activity (58). One possible explanation is that internet use alone may not be sufficiently linked to higher physical activity levels unless it is accompanied by social support, health information, or motivational engagement. In the present study, when social support and intrinsic motivation were included in the analysis, the direct association between internet use and physical activity was no longer statistically significant. This pattern suggests that the relationship between internet use and physical activity may operate primarily through psychosocial pathways rather than through a simple direct one. These findings suggest that psychosocial mechanisms, particularly social support and intrinsic motivation, may better explain differences in physical activity participation among older women than Internet use alone. These findings also suggest that future digital physical activity interventions targeting older women should not rely solely on providing online information but should also incorporate interpersonal support and motivational elements to promote sustained participation in physical activity.

Secondly, this study further suggests that social support is significantly associated with physical activity participation among older women. As a crucial external resource, social support may provide assistance when needed and create a favorable external environment for maintaining regular physical activity. Specifically, the adequacy of sports facilities, a positive exercise environment, material and financial support, and emotional support, all play important roles in motivating older women to engage in physical activity (59). Wang finds that older women who maintain frequent face-to-face or telephone contact with their children exhibit significantly higher rates of physical activity (60). Overall, the present findings indicate that social support is positively correlated with physical activity among older women. Furthermore, the results also indicate that internet use is positively associated with social support among older women. Prior research has shown that older women use internet applications such as Douyin, Tangdou, and Kuaishou to access exercise information and health resources; these applications may provide exercise information and health resources that support their participation in physical activity (61). Meanwhile, Internet use may expand older women’s social networks and facilitate communication with teammates, friends, and family members, thereby increasing opportunities to receive exercise-related encouragement and support (62). Subrahmanyam et al. (43) also indicate that through the internet, older adults can keep in touch with geographically dispersed relatives and friends, participate in online communities that share interests, maintain close communication with their children and grandchildren, and establish new connections with the outside world (63). This not only strengthens older adults’ ties to external social networks but also enhances communication within the family, helping them obtain intergenerational, instrumental, and emotional support (64). These findings suggest that internet use may be a channel for older women to obtain social interaction and support related to physical activity (65). Therefore, our study findings suggest that social support mediates the association between internet use and physical activity among older women. This expands on previous research by indicating that social support may not only be associated with physical activity but also serve as an interpersonal pathway linking digital engagement to regular physical activity participation. Given that physical activity is important for mobility, balance, and musculoskeletal health in later life, digital or remote physical activity programs for older women may benefit from integrating online peer groups, supportive family communication, and community-based digital support—rather than relying solely on exercise videos or health information.

Thirdly, this study reveals that intrinsic motivation mediates the association between internet use and physical activity. The results indicate that intrinsic motivation is positively associated with physical activity among older women, consistent with previous research (53, 54). This finding is also theoretically aligned with self-determination theory, which emphasizes the role of autonomy, competence, and relatedness in sustaining behavior. Previous research has shown that older women with stronger digital skills and more online experience may be better at identifying useful health and exercise information, which can help enhance their confidence and sense of self-efficacy (60). In the context of this study, intrinsic motivation may help explain why the association between internet use and physical activity is indirect rather than direct. This research indicates that internet use is positively associated with intrinsic motivation among older women, and stimulating this motivation can further encourage their participation in physical activity. Existing research indicates that individual behavior is shaped by interactions between environmental and individual factors, with motivation as a key psychological factor associated with participation in physical activity (46). For example, Spence and Lee found that intrinsic motivation has a positive effect on physical activity (27). Older women who have strong intrinsic motivation and enjoy physical activity are more likely to initiate and maintain physical activity habits over the long term (52). In summary, the findings support the hypothesis that intrinsic motivation mediates the association between internet use and physical activity among older women. This finding highlights the significance of motivational factors such as personalized feedback, autonomy-supportive guidance, remote coaching, and engaging exercise content, which may help older women translate their online health engagement into sustained physical activity.

Finally, an important finding of this study is that social support and intrinsic motivation sequentially mediated the association between internet use and older women’s physical activity. Specifically, internet use is associated with greater social support, which in turn is associated with stronger intrinsic motivation and higher levels of physical activity. This finding integrates Social Support Theory and Self-Determination Theory, indicating that interpersonal resources may help link digital engagement to intrinsic motivation for physical activity. This sequential pathway may be particularly important for older women, as their participation in physical activity is often influenced by gender- and age-related barriers. Existing research indicates that older women generally face multiple structural barriers when participating in physical activity. For instance, they may not receive enough encouragement and social support for physical activity, and they may also be more susceptible to age-related stereotypes, which may reduce their opportunities and motivation to participate in physical activities (66). Prior research has suggested that, as older women have to shoulder caregiving and household responsibilities in their later years, the time and energy they can devote to physical activity may be limited (67). Although this study did not directly measure these role-related factors, they may help explain why social support remains particularly crucial for older women to maintain physical activity. When individuals receive emotional support from family, peers, and the community, it may enhance their sense of self-efficacy and self-confidence, thereby increasing motivation to engage in physical activity (68, 69). This process may support intrinsic motivation and enhance behavioral autonomy, and persistence (70, 71). Family and peer support may represent important facilitators of older women’s participation in physical activity. Notably, perceived social support may be particularly important because it reflects older women’s subjective assessment of their existing social resources (72). Positive family interactions, peer encouragement, and community support may collectively foster a supportive environment that helps individuals cultivate and maintain physical activity. This may strengthen intrinsic motivation for exercise and facilitate long-term adherence and habit formation (73). In summary, the findings suggest that internet use is associated with physical activity among older women through a sequential mediating pathway involving social support and intrinsic motivation. This provides preliminary evidence that digital inclusion may promote physical activity among older women through psychosocial pathways, with potential implications for physical activity behaviors relevant to mobility, balance, and functional independence. Moreover, it may inform the design and implementation of digital physical activity interventions tailored to this population. However, this study did not directly measure musculoskeletal outcomes. Future longitudinal and intervention studies are needed to investigate whether using digital technologies to enhance social support and intrinsic motivation can support sustained engagement in physical activity and ultimately improve musculoskeletal health outcomes.

Despite a comprehensive exploration of the association between internet use and physical activity, as well as their potential psychosocial pathways, this study has several limitations. First, this study did not conduct stratified analyses by urban–rural residence, nor did it examine regional differences, which may have overlooked heterogeneity in internet access, digital skills, and usage contexts. In addition, other potentially relevant variables, such as health status, digital literacy, reasons for going online, family caregiving responsibilities, and access to fitness facilities, were not adequately captured, which may have introduced omitted-variable bias. Second, all variables were self-reported. Although common method bias was assessed and the results suggested that it was not a serious concern, its potential influence cannot be entirely ruled out. Third, the use of cross-sectional data limits the ability to draw causal inferences; therefore, future studies could adopt longitudinal or experimental designs to test causal mechanisms more rigorously. Fourth, there are limitations in measuring key variables. Physical activity was assessed through a single self-reported frequency item, which may not fully capture its multidimensional nature. Similarly, internet use was measured only by frequency, without distinguishing between different purposes such as health information retrieval, social interaction, or entertainment, which may obscure important differences. Moreover, this study focuses on intrinsic motivation but fails to adequately address other forms of motivation along the self-determination continuum, including identified regulation, introjected regulation, external regulation, and amotivation. Future research should employ more comprehensive, validated, and multidimensional measures of physical activity, internet use, and motivation. Fifth, although the findings may offer insights into musculoskeletal health promotion, this study did not directly measure musculoskeletal-related outcomes, such as muscle strength, balance, mobility, pain, fall risk, and functional independence. Therefore, the relevance of these findings to musculoskeletal health should be considered indirect and preliminary. Future longitudinal or intervention studies should examine whether digital technology-facilitated improvements in social support and intrinsic motivation can support sustained participation in physical activity and ultimately improve musculoskeletal-related functional outcomes. Finally, given the rapid pace of technological advancement, the data collected may not fully reflect the current internet usage patterns of older women. Future research may also incorporate qualitative methods, such as in-depth interviews and focus group discussions, to further explore the contextual implications and potential pathways underlying these associations.

## Conclusion

This study examined the association between internet use and physical activity among older women, as well as the serial mediating roles of social support and intrinsic motivation. The results demonstrate that social support and intrinsic motivation mediate the association between internet use and older women’s physical activity. Furthermore, social support and intrinsic motivation sequentially mediated the association between internet use and physical activity among older women. These findings suggest that efforts to improve older women’s access to the internet and digital engagement, expand their social support networks, and strengthen their intrinsic motivation may help support participation in physical activity. Although this study did not directly assess musculoskeletal outcomes, these findings may have indirect implications for musculoskeletal health promotion and active aging among older women.

## Data Availability

The original contributions presented in the study are included in the article/supplementary material, further inquiries can be directed to the corresponding authors.
